# Asymmetric Dimethylarginine Enables Depolarizing Spikes and Vasospasm in Mesenteric and Coronary Resistance Arteries

**DOI:** 10.1161/HYPERTENSIONAHA.123.22454

**Published:** 2024-01-16

**Authors:** Yu Y. Hanson Ng, Kim A. Dora, Hamish A.L. Lemmey, JinHeng Lin, James Alden, Lillian Wallis, Lucy Donovan, Oliver Shorthose, Fiona C. Leiper, James Leiper, Christopher J. Garland

**Affiliations:** 1 Department of Pharmacology, University of Oxford, United Kingdom (Y.Y.H.N., K.A.D., H.A.L.L., J. Lin, J.A., L.W., LD., O.S., C.J.G.).; 2 Institute of Cardiovascular and Medical Sciences, College of Medicine, Veterinary, and Life Sciences, University of Glasgow, United Kingdom (F.C.L., J. Leiper).

**Keywords:** cardiovascular diseases, coronary artery, endothelium, hypertension, ischemic heart disease

## Abstract

**BACKGROUND:**

Increased vasoreactivity due to reduced endothelial NO bioavailability is an underlying feature of cardiovascular disease, including hypertension. In small resistance arteries, declining NO enhances vascular smooth muscle (VSM) reactivity partly by enabling rapid depolarizing Ca^2+^-based spikes that underlie vasospasm. The endogenous NO synthase inhibitor asymmetric dimethylarginine (ADMA) is metabolized by DDAH1 (dimethylarginine dimethylaminohydrolase 1) and elevated in cardiovascular disease. We hypothesized ADMA might enable VSM spikes and vasospasm by reducing NO bioavailability, which is opposed by DDAH1 activity and L-arginine.

**METHODS:**

Rat isolated small mesenteric arteries and myogenic rat-isolated intraseptal coronary arteries (RCA) were studied using myography, VSM intracellular recording, Ca^2+^ imaging, and DDAH1 immunolabeling. Exogenous ADMA was used to inhibit NO synthase and a selective DDAH1 inhibitor, N^G^-(2-methoxyethyl) arginine, to assess the functional impact of ADMA metabolism.

**RESULTS:**

ADMA enhanced rat-isolated small mesenteric arteries vasoreactivity to the α_1_-adrenoceptor agonist, phenylephrine by enabling T-type voltage-gated calcium channel-dependent depolarizing spikes. However, some endothelium-dependent NO-vasorelaxation remained, which was sensitive to DDAH1-inhibition with N^G^-(2-methoxyethyl) arginine. In myogenically active RCA, ADMA alone stimulated depolarizing Ca^2+^ spikes and marked vasoconstriction, while NO vasorelaxation was abolished. DDAH1 expression was greater in rat-isolated small mesenteric arteries endothelium compared with RCA, but low in VSM of both arteries. L-arginine prevented depolarizing spikes and protected NO-vasorelaxation in rat-isolated small mesenteric artery and RCA.

**CONCLUSIONS:**

ADMA increases VSM electrical excitability enhancing vasoreactivity. Endothelial DDAH1 reduces this effect, and low levels of DDAH1 in RCAs may render them susceptible to endothelial dysfunction contributing to vasospasm, changes opposed by L-arginine.

**NOVELTY AND RELEVANCE**

**What Is New?**

A naturally occurring NO synthase inhibitor, asymmetric dimethylarginine, predisposes to vasospasm by switching small mesenteric and coronary resistance arteries into an electrically excitable state, while vasodilator capacity is preserved by endothelium-dependent hyperpolarization.

NO-dependent endothelial vasodilation was blocked by asymmetric dimethylarginine in coronary arter-ies but only partially inhibited in mesenteric resistance arteries.

Persistent NO-vasodilation correlated with greater dimethylarginine dimethylaminohydrolase-expression in rat-isolated small mesenteric arteries endothelium and sensitivity to dimethylarginine dimethylaminohy-drolase inhibition.

**What Is Relevant?**

NO is a potent vasodilator released by the endothelium but must also be considered in terms of its chronic ability directly to suppress arterial vasoreactivity.

Enhanced small artery vasoreactivity due in part to increased electrical excitability may be a significant feature of microvascular dysfunction.

**Clinical/Pathophysiological Implications?**

Coronary microvascular dysfunction is a major con-tributing factor in ischemic heart disease. The underly-ing mechanisms are complex and poorly understood.

Recognizing loss of NO as a key feature of coronary microvascular dysfunction that may arise from endoge-nous accumulation of endothelial asymmetric dimethylarginine suggests chronic intervention to protect NO signaling should be given serious consideration.

Possibilities for pharmacological intervention include the use of selective T-type voltage-gated calcium channel blockers to reduce vascular smooth muscle electrical excitability, outcompeting endothelial accumulation of asymmetric dimethylarginine with L-arginine or sustaining cyclic guanosine monophosphate levels with phosphodiesterase inhibitors.

Reduced bioavailability of NO is ubiquitous in cardiovascular disease (CVD) and aging. NO bioavailability reflects a variety of factors, including the expression and activity of eNOS (endothelial NO synthase/NOS [NO synthase] 3). NOS activity is suppressed in vivo by asymmetric dimethylarginine (ADMA), formed by posttranslational methylation of protein arginine residues followed by proteolysis and release into the plasma. ADMA competes with L-arginine binding to eNOS and is thought to be the major endogenous modulator of NO synthesis, as plasma concentrations are 10× higher than the other asymmetric methylarginine, N^G^-monomethyl L-arginine. These inhibitors block all 3 forms of NOS, but plasma contains a third product of posttranslational methylation, symmetrical dimethylarginine, which does not directly inhibit NO synthesis.^1,2^

ADMA blocks the release and action of NO in a variety of in vitro preparations.^3–5^ The possibility it suppresses NO synthesis in vivo was suggested when elevated plasma levels were found in patients with chronic renal failure.^4^ Subsequently, raised plasma ADMA has been associated with CVD in numerous animal and human studies, including spontaneously hypertensive rats and patients with hypertension. In the latter, increased forearm blood flow to acetylcholine (ACh) was reduced, indicating loss of endothelial cell (EC) function that was restored by L-arginine coinfusion.^6–9^ Furthermore, in healthy volunteers low-dose ADMA infusion increased mean blood pressure and systemic vascular resistance.^10^ Overall, elevated ADMA levels strongly correlate with a range of cardiovascular risk factors including hypertension and increased morbidity and mortality in both myocardial infarction and stroke.^2,11–13^

Reduced NO bioavailability causes vascular hyperreactivity due in part to loss of vasodilator capacity to basal and stimulated release of NO. We recently demonstrated that block of NO synthesis with N^G^-nitroarginine methyl ester (L-NAME), or disruption of the vascular endothelium, dramatically increases VSM reactivity.^14^ The VSM cells switch on electrical excitability as latent T-type VGCCs are activated. These channels trigger depolarizing action potential-like Ca^2+^ spikes that also recruit L-type VGCCs leading to vasospasm.

The current study had 2 aims. First, to investigate whether, like L-NAME, the endogenous eNOS-inhibitor ADMA enables depolarizing spikes and vasospasm in both nonmyogenic rat-isolated small mesenteric arteries (RMAs) and myogenically active rat-isolated intraseptal coronary arteries (RCAs). Functional clarification is important, as nitro-arginine inhibitors such as L-NAME are reported to be more potent than methylated arginine derivatives.^15,16^ Second, to investigate whether endogenous metabolism by DDAH (dimethylarginine dimethylaminohydrolases) influences the functional impact of ADMA on small artery vasoreactivity. In the aorta, inhibition of DDAH raised the level of endogenous ADMA and enhanced vasoconstriction to phenylephrine.^17^ Our data show ADMA, like L-NAME, does enable depolarizing spikes, enhancing vasoreactivity. They also suggest the functional influence of DDAH1 varies between small arteries, as ADMA failed to block NO-mediated vasorelaxation in mesenteric resistance arteries unlike small coronary arteries. Overall, these data are consistent with the idea that the microvascular dysfunction underlying ischemic heart disease could in part reflect enhanced vasoreactivity following endogenous accumulation of ADMA. They also indicate that supplementation with L-arginine can reduce the impact of NO loss.

## METHODS

Male Wistar rat small mesenteric and intraseptal coronary arteries were mounted in a Mulvany-Halpern myograph to record isometric tension with simultaneous measurement of membrane potential or VSM intracellular Ca^2+^ events or in a pressure myograph.^18,19^ Arteries were then fixed in situ for immunohistochemistry. Data were analyzed using Microsoft Excel 2011 (Microsoft Corporation) and GraphPad Prism Software (v10.0, GraphPad Software).^20,21^ More detail is found in the Supplement Material, Expanded Materials and Methods. Data that support the findings of this study are available from the corresponding author upon reasonable request.

## RESULTS

### Action Potential-Like Spikes and Enhanced Vasoconstriction in RMA With ADMA

**Figure 1.**
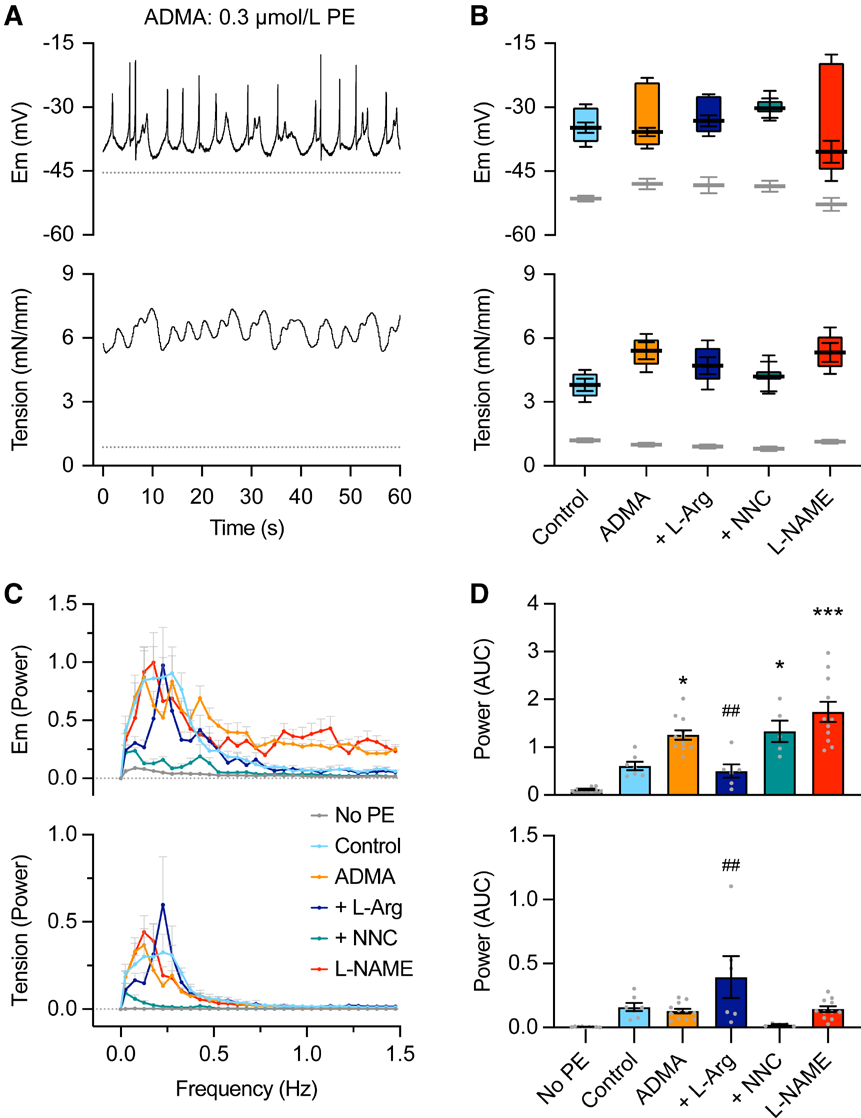
**Using asymmetric dimethylarginine (ADMA) to inhibit NO synthase in mesenteric arteries enables rapid depolarizing spikes and vasospasm on stimulation with phenylephrine (PE), an effect prevented by either L-arginine (L-Arg) or the T-type voltage-gated calcium channels (VGCC) blocker, NNC 55-0396 (NNC). A**, PE 0.3 µmol/L stimulated transient vascular smooth muscle (VSM) spikes and enhanced vasoconstriction with 300 µmol/L ADMA present. **B**, Box-plot summarizing the amplitude of depolarizing spikes and vasoconstriction, mean±SEM for maximum and minimum values of mV and mN/mm with sample mean. Membrane potential and tension before and in the presence of 3 µmol/L PE (control, n=9), 0.3 µmol/L PE with 300 µmol/L ADMA (n=12), 0.3 µmol/L PE with ADMA+1 mmol/L L-Arg (n=5), 0.3 µmol/L PE with ADMA+0.3 µmol/L NNC 55-0396 (n=5) and 0.3 µmol/L PE + 100 µmol/L L-NAME (n=11). Note 10-fold lower (PE) compared with control for similar depolarization/vasoconstriction with ADMA or L-NAME present. **C**, Fourier transform showing mean power of waveform during depolarization/spikes (**upper**) and associated vasoconstriction (**lower**); No-PE (n=9), PE (control, n=7), PE in the presence of ADMA (n=12), ADMA+L-Arg (n=6), ADMA+NNC (n=5) or with L-NAME rather than ADMA present (n=11). **D**, Mean power from each waveform in **C**. One-way ANOVA with Bonferroni multiple comparisons. Gray dotted lines indicate level pre-PE and colors in **C** and **D** match **B**. *P<0.05 vs control, ***P<0.001 vs control, ^##^*P*<0.01 vs ADMA. AUC indicates area under curve; and Em, VSM membrane potential.

Blocking NO synthase with 300 µmol/L ADMA did not alter either the resting membrane potential or tone of RMA smooth muscle cells (pre-ADMA, −51.4±0.7 mV, 1.2±0.1 mN/mm; n=9 and with ADMA, −48.0±1.2 mV, 1.0±0.1 mN/mm; n=12, *P*>0.05; Figure 1A and 1B). However, ADMA (300 µmol/L but not 100 µmol/L; Figure S1A) increased vasoreactivity, so previously ineffective concentrations of the α_1_-adrenoreceptor agonist phenylephrine (PE), 0.1 to 0.8 µmol/L, evoked vasoconstriction of similar magnitude to 1 to 3 µmol/L PE pre-ADMA (Figure S1A and S1B). Furthermore, in the presence of ADMA, PE induced rapid depolarizing spikes and chaotic vasomotion sensitive to the T-type VGCC blocker 0.3 µmol/L NNC 55-0396. The effect of ADMA was equivalent to the effect of 100 µmol/L L-NAME (Figure 1A and 1B). L-arginine (1 mmol/L) prevented or reversed the effect of ADMA, in some cases, reestablishing vasomotion to PE. The latter gave the most prominent waveform following Fourier analysis (Figure 1C and 1D).

### NO-Mediated Vasorelaxation to ACh Persists With ADMA in RMA

**Figure 2.**
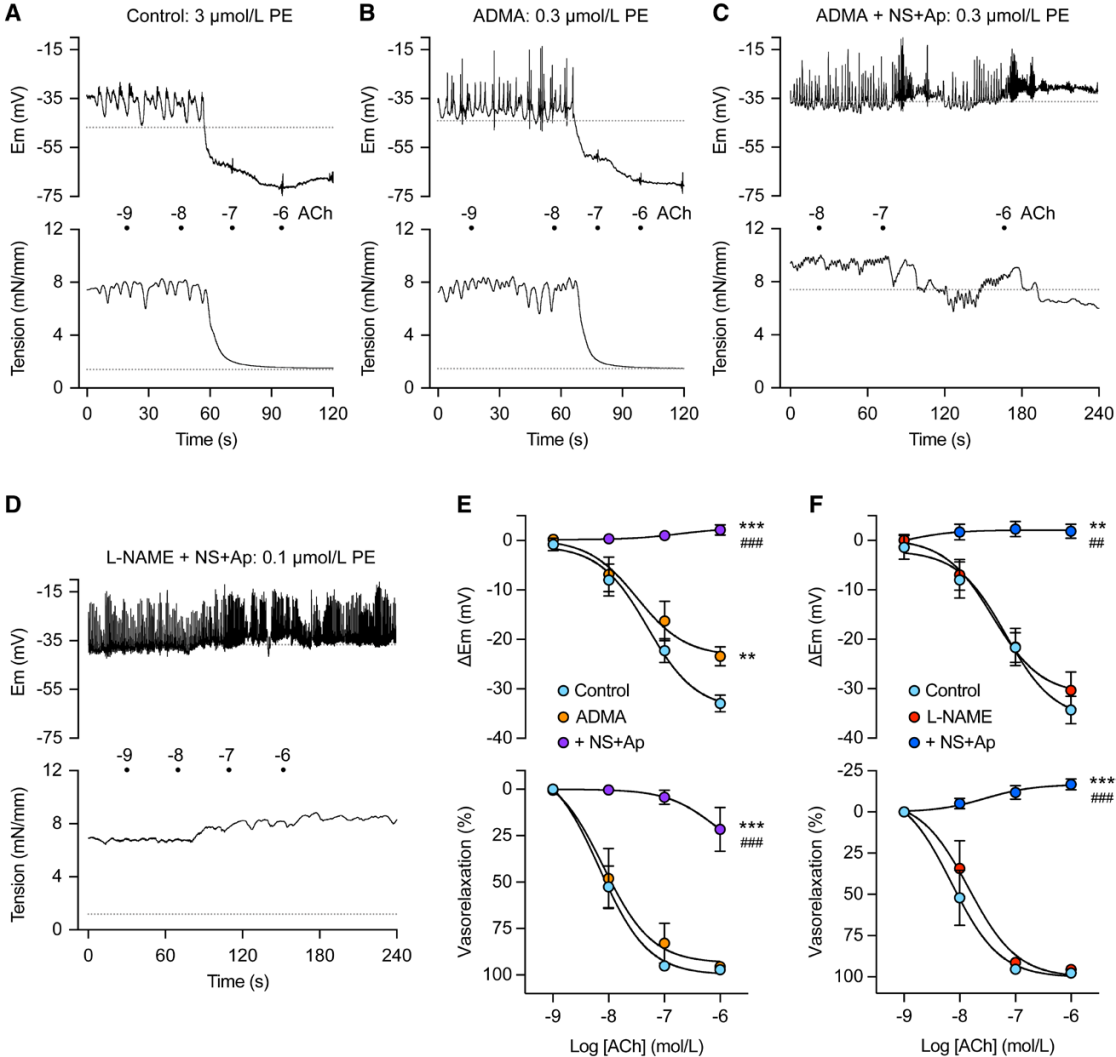
**Endothelium-dependent vasorelaxation to acetylcholine (ACh) persists in the presence of asymmetric dimethylarginine (ADMA) but not N^G^-monomethyl L-arginine methyl ester (L-NAME) during block of endothelium-dependent hyperpolarization (EDH) in mesenteric arteries. A**, Control hyperpolarization (**upper**) and vasorelaxation (**lower**) to cumulative ACh during 3 µmol/L PE-induced depolarization and vasoconstriction. **B**, Repeat after incubation with 300 µmol/L ADMA and 10-fold less PE (0.3 µmol/L) to achieve similar depolarization and vasoconstriction. **C**, After additional incubation with 1 µmol/L NS6180 and 0.1 µmol/L apamin to block endothelial cell K_Ca_3.1 and K_Ca_2.3 responsible for EDH. **D**, Artery exposed to 100 µmol/L L-NAME, 1 µmol/L NS6180, and 0.1 µmol/L apamin, followed by cumulative ACh during stimulation with 0.1 µmol/L PE. Gray dotted lines indicate level pre-PE. **E**, Summary hyperpolarization (**top**) and vasorelaxation (**lower**) to cumulative (ACh) during stimulation with 0.3 µmol/L PE (control, blue circles, n=9) repeated in the presence of 300 µmol/L ADMA (n=6), then the additional presence of 1 µmol/L NS6180 and 0.1 µmol/L apamin (n=5). **P<0.01 vs control, ***P<0.001 vs control, ^###^*P*<0.001 vs ADMA; 1-way ANOVA with Bonferroni multiple comparisons. **F**, ACh (control, n=5) then with 100 µmol/L L-NAME (n=5), then the additional presence of 1 µmol/L NS6180 and 0.1 µmol/L apamin (n=5). **P<0.01 vs control, ***P<0.001 vs control, ^##^*P*<0.01 vs L-NAME, ^###^*P*<0.001 vs L-NAME; RM 1-way ANOVA with Bonferroni multiple comparisons.

In PE-stimulated arteries with either 300 µmol/L ADMA or 100 µmol/L L-NAME present to block NO synthase, ACh evoked concentration-dependent smooth muscle hyperpolarization and vasorelaxation (an endothelium-dependent hyperpolarization (EDH) response; Figure 2A, 2B, 2E, and 2F). Endothelium-dependent mesenteric artery vasorelaxation is mediated by the parallel action of both NO and EDH, the latter generated by dual activation of EC SK_Ca_ and IK_Ca_ channels. Subsequent block of EDH with 1 µmol/L NS6180 (blocks EC IK_Ca_ channels, K_Ca_3.2^22^) combined with 0.1 µmol/L apamin (blocks endothelial SK_Ca_ channels, K_Ca_2.1) abolished EDH hyperpolarization and vasorelaxation in the presence of L-NAME. However, vasorelaxation persisted with ADMA and NS6180/apamin present, even though hyperpolarization was abolished (Figure 2C through 2F). Subsequent addition of the K_ATP_ channel activator, 5 µmol/L levcromakalim stimulated hyperpolarization (to −72±1.0 mV) and complete relaxation (to 1.2±0.2 mN/mm^−1^), n=5.

### N^G^-(2-Methoxyethyl) Arginine Abolishes While L-Arginine Rescues ACh Vasorelaxation in the Presence of ADMA

**Figure 3.**
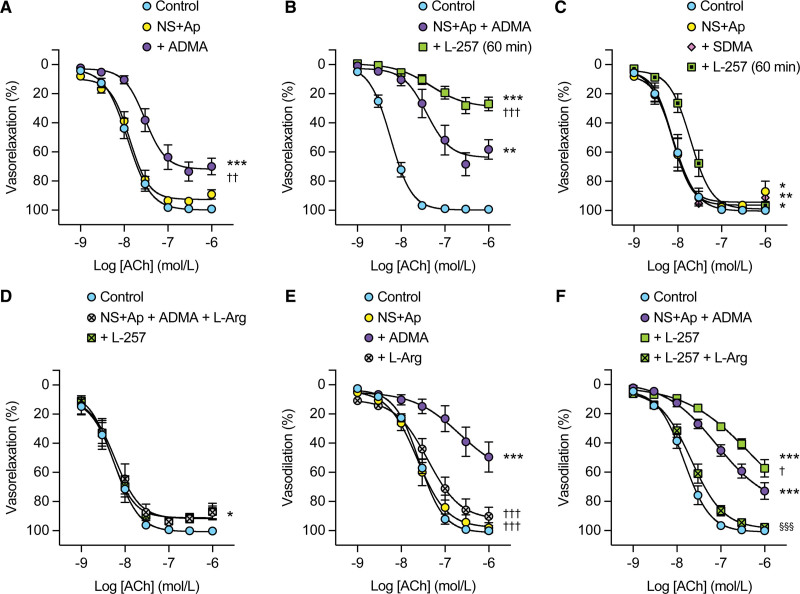
**Persistent acetylcholine (ACh) vasorelaxation in mesenteric arteries during asymmetric dimethylarginine (ADMA) exposure and endothelium-dependent hyperpolarization (EDH) block is further suppressed by the DDAH1 (dimethylarginine dimethylaminohydrolase 1) inhibitor, L-257 (N^G^-[2-methoxyethyl]-L-arginine), with inhibition reversed by L-arginine.** A, Summary data with no change from control (n=10) with 1 µmol/L NS6180 (NS) and 0.1 µmol/L apamin (Ap) present to block EDH (n=10), but inhibition on subsequent exposure to 300 µmol/L ADMA (n=10). ***P<0.001 vs control, ^††^*P*<0.01 vs NS6180 (NS + Ap). **B**, Residual ACh vasorelaxation in the presence of 1 µmol/L NS, 0.1 µmol/L Ap, and 300 µmol/L ADMA (n=7) was further diminished by subsequent addition of 100 µmol/L L-257 (60-minute exposure, n=7). **P<0.01 vs control, ***P<0.001 vs control, ^†††^*P*<0.001 vs NS+Ap+ADMA. **C**, ACh alone (control, n=6) and with NS and Ap (n=6), then in combination with 300 µmol/L symmetrical dimethylarginine (SDMA; n=6), and addition of L-257 (n=6). *P<0.05 vs control, **P<0.01 vs control. **D**, 1 mmol/L L-Arg (n=7) prevents inhibition of ACh vasorelaxation with a combination of 300 µmol/L ADMA, 1 µmol/L NS6180, 0.1 µmol/L apamin, but not in the presence of 100 µmol/L L-257 (n=7, *P<0.05 vs control). **E**, Vasodilation (pressure myography, control, n=6) was unaffected by 1 µmol/L NS6180 and 0.1 µmol/L apamin (n=6) but inhibited by additional 300 µmol/L ADMA (n=6, ***P<0.001 vs control), an effect reversed by 1 mmol/L L-Arg (n=6, ^†††^*P*<0.001 vs NS+Ap+ADMA). **F**, Vasodilation (n=9) in pressurized arteries inhibited by 300 µmol/L ADMA, 1 µmol/L NS, and 0.1 µmol/L Ap (n=9, ***P<0.001 vs control), and further addition of 100 µmol/L L-257 (n=9, ^†^*P*<0.05 vs NS+Ap+ADMA) and reversal by 1 mmol/L L-Arg (n=9, ^§§§^*P*<0.001 vs NS+Ap+ADMA+L-257). All experiments were paired; all statistical tests were RM 1-way ANOVA, with Bonferroni multiple comparisons.

Persistent RMA vasorelaxation to ACh during block of both EDH (with 1 µmol/L NS6180 and 0.1 µmol/L apamin) and NO synthesis using ADMA (300 µmol/L) was reduced by the subsequent addition of a selective DDAH1 inhibitor, 100 µmol/L L-257 (N^G^-[2-methoxyethyl] arginine)^23^ (Figure 3A and 3B). If ADMA was replaced by the inactive ADMA isomer, symmetric dimethylarginine (300 µmol/L), control vasorelaxation to ACh was not reduced even if L-257 was then added for 60 minutes (Figure 3C).

L-arginine (1 mmol/L) prevented the block of ACh vasorelaxation with NS6180, apamin, and ADMA combined, even after an additional 60-minute exposure to L-257 (Figure 3D).

A similar profile was obtained in pressurized RMA with ADMA. Circa 50% ACh-vasodilation persisted during block of EDH (with NS6180 and apamin) and eNOS, the latter with 300 µmol/L ADMA. Loss of vasodilation was augmented in the presence of L-257. As with wire myography, ACh vasodilation could be protected on incubation with 1 mmol/L L-arginine (Figure 3E), even when DDAH1 was inhibited with L-257 (Figure 3F).

**Figure 4.**
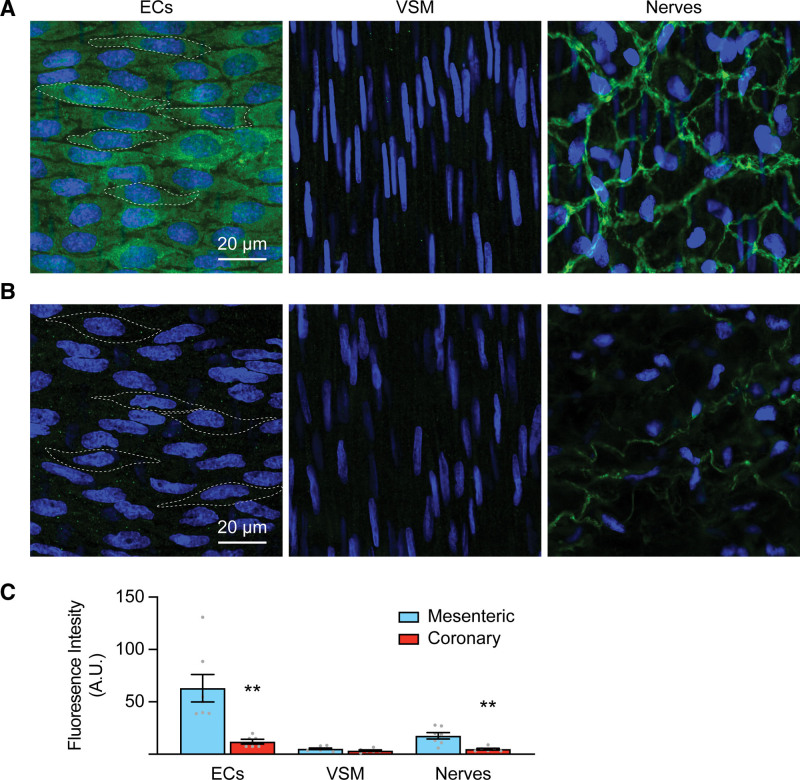
**Greater DDAH1 (dimethylarginine dimethylaminohydrolase 1) expression in mesenteric compared with coronary artery endothelial cells (ECs).** Representative immunohistochemistry labeling for DDAH1 (green) in ECs (**left**), vascular smooth muscles (VSMs; **middle**), and perivascular nerves (**right**). **A**, Rat-isolated small mesenteric arteries, RMA, n=7 and **B**, rat-isolated intraseptal coronary arteries, RCAs, n=6; nuclei labeled blue, scale bar, 20 µm. **C**, Summary data of mean DDAH1 fluorescence intensity from the 5 outlined EC cells and whole field fluorescence for VSM/nerves. **P<0.01; unpaired *t* test.

Immunohistochemistry indicated DDAH1 in both mesenteric and coronary arteries (Figure 4A and 4B; negative control Figure S2A and S2B). Nonspecific bands were not detected with the DDAH1 antibody in Western blots of kidney or liver tissue, where DDAH1 is highly expressed (Figure S2C). DDAH1 expression was greater in RMA ECs and perivascular nerves (intensity ECs, 63.1±12.1 AU; intensity nerves, 17.7±3.1 AU, n=7) compared with RCA (12.1±2.2 AU, 4.9±0.9 AU, n=6, *P*<0.01). VSM DDAH1 expression was low in both arteries (Figure 4C).

### Action Potential-Like Spikes and Enhanced Vasoconstriction With ADMA in RCA

**Figure 5.**
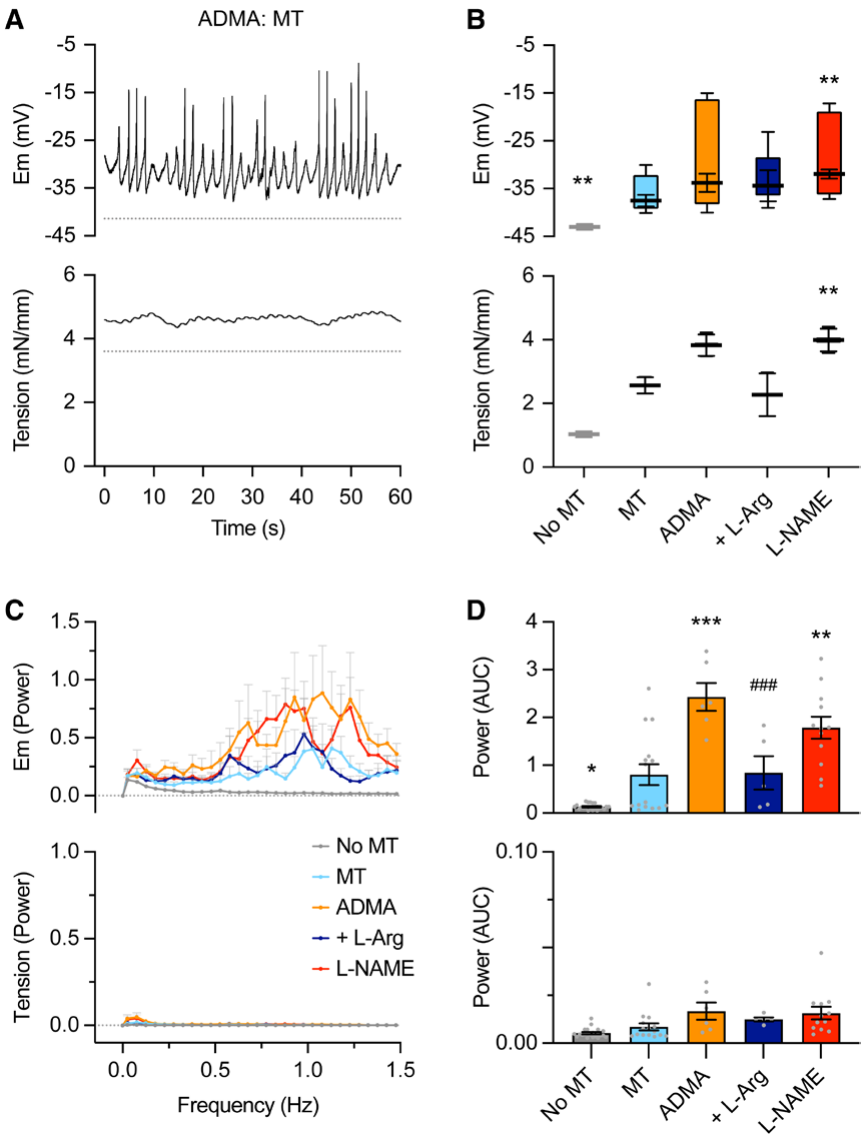
**Rapid depolarizing spikes and vasospasm during exposure to asymmetric dimethylarginine (ADMA) reversed by L-arginine in coronary arteries. A**, Representative traces of vascular smooth muscle (VSM) spikes (**top**) and enhanced vasoconstriction (**lower**) with 300 µmol/L ADMA. Gray dotted lines, membrane potential and myogenic tone before ADMA. **B**, Box plot summarizing the amplitude of depolarizing spikes and vasoconstriction as mean±SEM for the maximum and minimum values of mV and mN/mm with sample mean. Membrane potential and tension before myogenic tone developed (n=22), with myogenic tone (MT, n=19), with 300 µmol/L ADMA (n=6), ADMA+1 mmol/L L-Arg (n=5), 100 µmol/L N^G^-monomethyl L-arginine methyl ester (L-NAME; n=12). **P<0.01 vs MT. **C**, Fourier transform showing mean power of waveform during depolarization/spikes (**upper**) and associated vasoconstriction (**lower**); n, as above in **B. D**, Mean power from each waveform in **C**. *P<0.05 vs MT, **P<0.01 vs MT, ***P<0.001 vs MT, ^###^*P*<0.05 vs ADMA. All statistical tests were 1-way ANOVA with Bonferroni multiple comparisons, except for tension in **D**, where Kruskal-Wallis test with Dunn multiple comparisons was performed. AUC indicates area under curve; and Em, VSM membrane potential.

In contrast to RMA, around 50% of small coronary arteries spontaneously developed myogenic tone (without MT, 1.0±0.1 mN/mm; n=22 versus MT, 2.6±0.3 mN/mm; n=19, respectively) with VSM resting potentials of −43.0±0.6 mV, n=22 versus −37.5±1.3 mV, n=19 and small (circa 10 mV) depolarizing spikes in 10 of these arteries. ADMA 300 µmol/L depolarized VSM to −33.8±1.9 mV and increased vasoconstriction to 3.8±0.3 mN/mm, n=6 (Figure 5A and 5B). These arteries now generated large depolarizing spikes (mean amplitude, 21.6±1.1 mV; maximum for individual spikes, 27.9±3.4 mV; mean frequency, 1.5±0.4 Hz). In separate experiments, similar frequency Ca^2+^ flashes were apparent increasing with ADMA (Figure S3, Video S1). L-NAME had a similar effect; vasoconstriction (to 3.2±0.4 mN/mm, n=12) and depolarization (to −31.9±1.0 mV, n=12) with the appearance of rapid depolarizing spikes (mean amplitude, 17.0±2.0 mV, 1.0±0.1 Hz, maximum, 25.4±3.3 mV, n=12). L-arginine (1 mmol/L) reduced both spike amplitude and associated vasoconstriction with ADMA to control levels (*P*>0.05; Figure 5B). Waveform analysis of membrane potential changes illustrates both variability in spike frequency, the increased power with ADMA and L-NAME, and reversal with L-arginine, summarized in Figure 5D. Tension analysis reflected the sustained vasoconstriction at each level.

### NO-Vasorelaxation in RCA Is Abolished by ADMA or L-NAME

**Figure 6.**
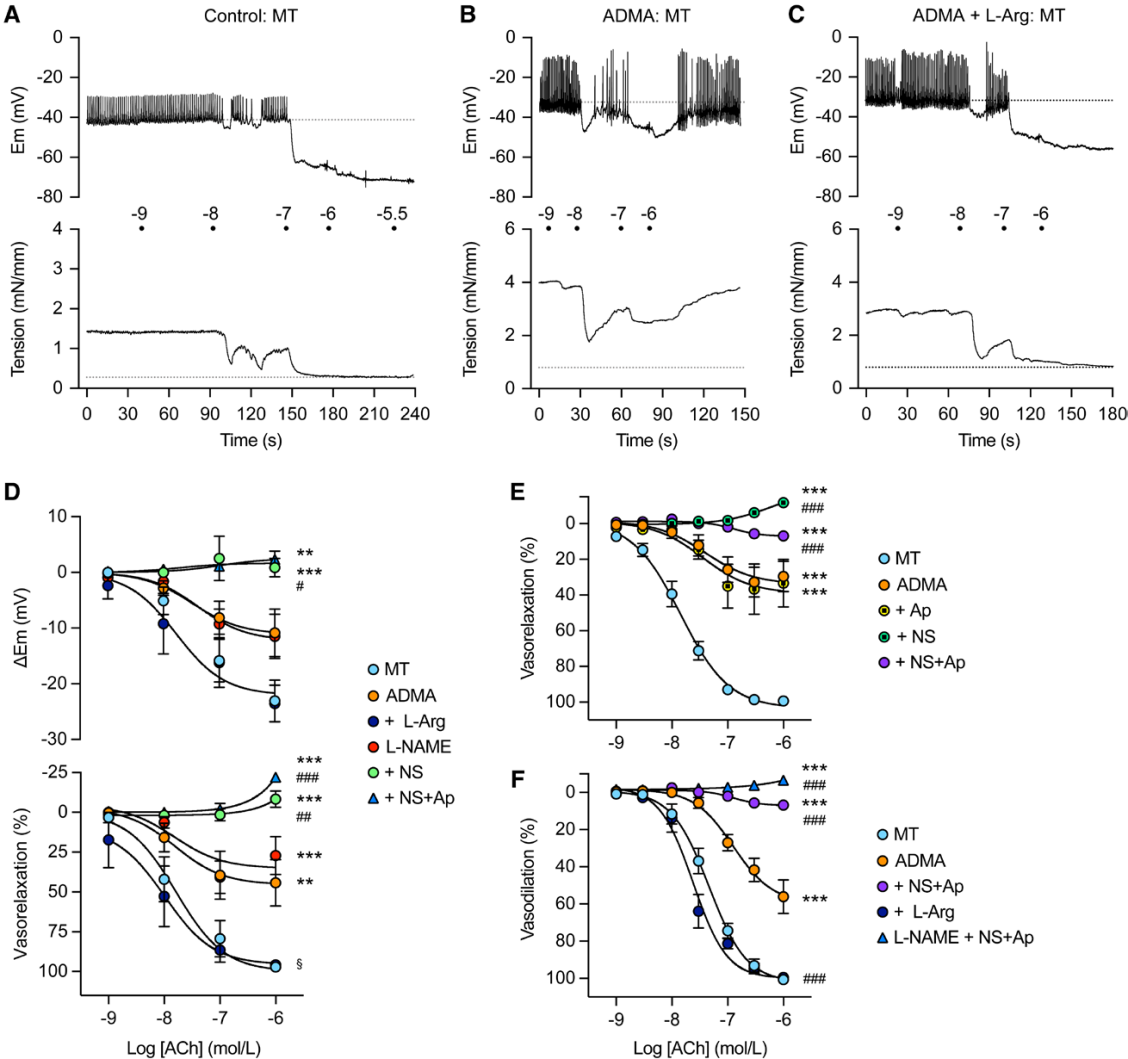
**Endothelium-dependent vasorelaxation in coronary arteries is abolished with NO synthase and IK_Ca_ inhibition. A**, Control hyperpolarization (**upper**) and vasorelaxation (**lower**) to cumulative (acetylcholine [ACh]) during myogenic tone (MT). Gray dotted lines, pre-MT. **B**, Asymmetric dimethylarginine (ADMA) 300 µmol/L enhanced depolarizing spikes and diminished hyperpolarization and vasorelaxation, which became transient. **C**, In the additional presence of 1 mmol/L L-Arg, hyperpolarization and vasorelaxation were restored. **D**, Mean±SEM summarizing hyperpolarization (**upper**) and vasorelaxation (**lower**) to cumulative ACh against MT (control, n=8), with 300 µmol/L ADMA (n=7), 300 µmol/L ADMA+1 mmol/L L-Arg (n=5), 100 µmol/L L-NAME (n=13), L-NAME+1 µmol/L NS (NS6180; n=6), L-NAME+NS6180+0.1 µmol/L Ap (apamin; n=6). **P<0.01 vs MT, ***P<0.001 vs MT, ^#^*P*<0.05 vs L-NAME, ^##^*P*<0.01 vs L-NAME, ^###^*P*<0.001 vs L-NAME, ^§^*P*<0.05 ADMA+L-Arg vs ADMA. **E**, Vasorelaxation to cumulative ACh against MT (control, n=12), with 300 µmol/L ADMA (n=12), ADMA+1 µmol/L NS6180 (n=7), ADMA+0.1 µmol/L apamin (n=7), ADMA+NS6180+0.1 µmol/L apamin (n=7). ***P<0.001 vs MT, ^###^*P*<0.001 vs ADMA. **F**, Vasodilation to ACh (pressure myography, MT, n=12), with 300 µmol/L ADMA (n=12), ADMA+1 mmol/L L-Arg (n=8), ADMA+1 µmol/L NS6180+0.1 µmol/L apamin (n=7), 100 µmol/L L-NAME+NS6180+Ap (n=6). ***P<0.001 vs control, ^###^*P*<0.001 vs ADMA. All statistical tests were 1-way ANOVA with Bonferroni multiple comparisons.

ADMA 300 µmol/L inhibited hyperpolarization and vasorelaxation to ACh, an effect reversed by 1 mmol/L L-arginine (Figure 6A and 6C, summaries Figure 6D, pressurized arteries Figure 6F). This concentration of L-arginine reversed vasoconstriction induced by 300 µmol/L ADMA but not 100 µmol/L L-NAME (Figure S4). Lower concentrations (10 and 30 µmol/L) of ADMA significantly increased myogenic tone and 30 µmol/L ADMA inhibited, but did not block, ACh vasorelaxation (Figure S1C and S1D).

In the presence 300 µmol/L ADMA, block of EDH with NS6180 and apamin abolished ACh vasorelaxation in RCA (Figure 6E and 6F). A similar profile was obtained using 100 µmol/L L-NAME, which was augmented by EDH block (Figure 6D and 6F; Figure S5). Levcromakalim 1 µmol/L hyperpolarized and completely relaxed these arteries (with ADMA present to −70.8±1.1 mV, 0.6±0.2 mN/mm^−1^, n=6; with L-NAME to −69.4±2.1 mV, 0.8±0.2 mN/mm^−1^, n=6, respectively).

## DISCUSSION

We show the endogenous NO synthase inhibitor, ADMA, predisposes small resistance arteries to vasospasm by inducing a hyperexcitable state due to depolarizing action potential-like spikes in the VSM, rather than loss of NO-dependent vasorelaxation. The latter is sustained by EDH, although reduced in the RCA where NO contributed to EDH. Once EDH was blocked the sensitivity to ADMA varied between RMA and RCA. NO-vasorelaxation was abolished in RCA, but only partially inhibited in RMA where persistent NO-vasorelaxation appeared to reflect ADMA metabolism by DDAH1.

Block of endothelial NO synthase with synthetic arginine derivatives such as L-NAME markedly increases vascular reactivity. We recently discovered increased vasoreactivity is due to increased VSM electrical excitability, rather than reduced endothelial vasodilation. Raised electrical excitability enabled previously quiescent VSM to generate Ca^2+^-based depolarizing spikes with vasospasm. The spikes were reminiscent of action potentials, apart from a variable amplitude, and due to recruitment of latent T-type-VGCCs.^14^ We now show the endogenous methylarginine, ADMA can induce a similar change in small arteries. Previous data suggest endogenous methylarginines, N^G^-monomethyl L-arginine and ADMA, are not always as potent as widely used synthetic NO inhibitors, so this is an important observation.^15,16,24^ The interaction between ADMA and NO synthase is reversible, and our data are consistent with this, as 1 mmol/L L-arginine prevented or reversed increased VSM electrical activity, vasoreactivity, and the loss of endothelium-dependent ACh vasorelaxation with 300 µmol/L ADMA. Interestingly, RCAs were far more sensitive to ADMA than RMA, consistent with lower EC-DDAH1 expression. Importantly, increased myogenic tone with 10 µmol/L ADMA was reversed with 30 µmol/L L-arginine (Figure S1C), consistent with ≈3:1 ratio for reversing ADMA.

Small artery hyperexcitability due to VSM electrical activity may represent an important component of microvascular dysfunction. Increased RhoA/Rho kinase signaling has been linked to vasospasm in the coronary microvasculature,^25^ so electrical activity causing calcium influx would interact synergistically with VSM-sensitization. These mechanisms are likely linked, as PKG phosphorylation prevents RhoA translocating to the cell membrane, so as well as uncovering latent T-type VGCCs triggering Ca^2+^-spikes, loss of NO will enhance Rho kinase signaling.^26–28^

Loss of endothelium-dependent vasorelaxation is thought central to the increased vasoreactivity with declining NO bioavailability. In small resistance arteries, endothelium-dependent vasorelaxation reflects the parallel influence of hyperpolarization (EDH) and NO release. The former is due to hyperpolarizing current generated by small conductance calcium-activated potassium channel (SK_Ca_) and intermediate conductance calcium-activated potassium channels (IK_Ca_) in channels in the endothelium, and block of both channel types is usually necessary to block EDH.^29^ In contrast to most small arteries, EDH in RCA was abolished by the IK_Ca_ blocker NS6180 alone. Importantly, NO also contributed to endothelial-hyperpolarization in RCA, as L-NAME and ADMA each reduced ACh hyperpolarization and vasorelaxation. This inhibitory component was prevented by L-arginine. In RMA, although EDH loss enhanced depolarization and vasoconstriction to the adrenergic agonist PE, it did not enable spike potentials, as NO was still available to suppress T-type VGCCs.^14^ In contrast with L-NAME, we show ADMA failed to block RMA NO-mediated vasorelaxation, although it did increase VSM electrical excitability. This suggests less NO is required for vasorelaxation than to suppress Ca^2+^-based depolarization.

ADMA is metabolized by intracellular DDAH, which has 2 isoforms DDAH1 and DDAH2.^17,23^ DDAH2 cannot metabolize ADMA, but metabolism by DDAH1 has a significant influence on arterial function, as block with S-2-amino-4(3-methylguanidino)butanoic acid (412W) induced progressive vasoconstriction in rat aorta, which was reversed by exogenous L-arginine. 412W also reversed the loss of endothelium-dependent (NO) vasorelaxation in human saphenous artery.^17,30^ Both effects demonstrate continual turnover of methylarginines, with DDAH1 ensuring the intracellular ADMA accumulation is not normally sufficient to block NO synthesis. We used the recently developed and selective DDAH1 inhibitor, L-257, which binds within the active site of the enzyme elevating ADMA sufficiently to inhibit NO signaling.^23^ In RMA, the ability of L-257 to inhibit ADMA-resistant ACh vasorelaxation suggests that ADMA DDAH1-metabolism normally protects NO synthase activity. This profile was similar in wire-mounted and pressurized RMA, and 1 mmol/L L-arginine prevented the inhibitory action of ADMA±L-257. Although DDAH1 has specific affinity for ADMA (and N^G^-monomethyl L-arginine), it does not degrade L-NAME, consistent with the divergent effects obtained with each inhibitor in RMA.^31^ The profile of inhibition in RCA was different. Once EDH was blocked, either ADMA or L-NAME totally abolished ACh vasorelaxation. Low levels of DDAH1 will predispose RCA to ADMA accumulation, block of NOS and enhanced vasoreactivity, it also indicates methylarginine metabolism varies between vascular beds.

Overall, the present experiments show the endogenous methylarginine, ADMA can enhance small artery vasoreactivity in a similar way to the synthetic NO synthase inhibitor L-NAME. In both cases, increased vasoreactivity is due to VSM electrical excitability developing on loss of NO. However, as only male rats were used in the present study, to limit inter-sex variability, these conclusions require verification in females. The importance of our observations is contextualized by a large literature supporting a fundamental role for ADMA in CVD, when plasma levels increase from low to mid micromolar concentrations and ADMA is considered an independent predictor of morbidity and mortality.^2,4^ While this range is close to NO synthase K_i_, plasma concentrations of L-arginine are greater (>100 µmol/L and mmol/L intracellularly) questioning the importance of ADMA in CVD. However, arginine supplementation enhances NO bioavailability, referred to as the “arginine paradox”. Enhanced NO is attributed to arginine overcoming constitutive NOS inhibition by ADMA. Close to the site of synthesis, intracellular concentrations of ADMA will be far greater than in plasma, which may explain these observations.^23^

Elevated levels of ADMA not only block NO synthase but also increase the production of reactive oxygen species, reducing NO bioavailability.^32–34^ Thus, ADMA accumulation may be a major contributor to the decline in NO causing microvascular dysfunction, including coronary microvascular dysfunction. In vasospastic angina, reduced amino acid transporter activity has been linked to ADMA accumulation, eNOS uncoupling, and systemic endothelial dysfunction.^35^ Coronary microvascular dysfunction is now a recognized cause of ischemic heart disease that precedes and predicts obstructive coronary artery disease.^36–38^ Enhanced vasoreactivity in coronary microvascular dysfunction reflects both functional and structural abnormalities and is associated with dysfunction in other parts of the circulation, such as human digital and gluteal arteries.^39–41^ Widespread dysfunction across the vasculature is consistent with declining endothelial NO bioavailability.^42^

## PERSPECTIVES

In large arteries, NO is the predominant endothelium-dependent vasodilator and the increased vasoreactivity that develops in CVD has been ascribed to loss of NO-vasodilator capacity. The VSM cells in small resistance arteries have a greater density of L-type VGCCs, and vasodilation is dominated by EDH-linked changes in membrane potential. As a result, EDH can sustain endothelium-dependent vasodilation in the absence of NO, although less so in coronary arteries where NO contributes to EDH. As well as vasodilation, we show NO directly suppresses VSM reactivity and reduce bioavailability with the naturally occurring NO synthase inhibitor ADMA enables spontaneous depolarizing spikes and vasospasm in small coronary arteries. Our data also suggest the capacity of DDAH1 to metabolize ADMA is less in RCA than other parts of the circulation, potentially predisposing these arteries to increased vasoreactivity/vasospasm as ADMA accumulates. It is, therefore, important to consider increased vasoreactivity as a change in the VSM, not simply as a loss of NO-vasodilator capacity. As NO chronically suppresses VSM excitability, opposing or reversing NO loss may offer an effective approach to address coronary microvascular dysfunction and possibly the development of CVD. We previously suggested selective T-type VGCC block as a strategy to counter enhanced VSM electrical activity, without abolishing the myogenic tone necessary for blood flow autoregulation as the latter is underpinned by L-type VGCCs.^14^ An alternative/additional possibility is to enhance/protect NO signaling with L-arginine supplementation or raise cyclic guanosine monophosphate with selective phosphodiesterase 5 inhibitors. Both would avoid long-term use of nitrates to generate NO and the associated complications from tolerance and undesirable side effects that include nitrosative stress.^43^

## ARTICLE INFORMATION

### Sources of Funding

This study was funded by BHF for 4-year DPhil Studentship FS/19/61/34900 (Y.Y. Hanson Ng), FS/18/63/34184 (L. Donovan); British Heart Foundation PG/19/36/34396, PG/20/10260, and PG/23/11496 (J. Lin and H.A.L. Lemmey); Leon and Iris Beghian Scholarship, Magdalen College (L. Wallis); British Heart Foundation infrastructure grant: IG/13/5/30431, British Heart Foundation Centre of Research Excellence: RE/13/1/30181.

### Disclosures

None.

## Supplementary Material
