# Supplementary material for: Asymmetric Dimethylarginine Enables Depolarizing Spikes and Vasospasm in Mesenteric and Coronary Resistance Arteries
Source: Hypertension. 2024 Jan 16;81(4):764–75. doi: 10.1161/HYPERTENSIONAHA.123.22454 (PMC10956675; doi:10.1161/HYPERTENSIONAHA.123.22454)
Supplement: Supplementary file 2 [file hyp-81-764-s002.pdf]

*** Short In Vivo Checklist**
AHA - Preclinical Animal Testing : Prevention of bias is important for experimental cardiovascular research. ***This short checklist must be completed, and the answers should be clearly presented in the manuscript as well.*** *The checklist will be used by reviewers and editors but will not be published. If a revision is requested, you will be required to complete at revision submission a more detailed checklist that will be published with the accepted article.*

This study involves animals:
Yes

Animals

Species, age, sex, strains, and sources of animals are described: Yes

Randomization

Randomization and allocation concealment were performed: Yes

Blinding

Blinding was performed: Yes

Inclusions and Exclusions (a)

Specific criteria for inclusions and exclusions are specified: Yes

Inclusions and Exclusions (b)

Criteria for inclusions and exclusions were set before the study: Yes

Reporting of Excluded Animals

All animals excluded after the randomization are reported: Yes

Statistical Methods

Statistical Methods are described: Yes

Date completed: 12/22/2023 15:51:45
User pid: 46325
