# Supplementary material for: Asymmetric Dimethylarginine Enables Depolarizing Spikes and Vasospasm in Mesenteric and Coronary Resistance Arteries
Source: Hypertension. 2024 Jan 16;81(4):764–75. doi: 10.1161/HYPERTENSIONAHA.123.22454 (PMC10956675; doi:10.1161/HYPERTENSIONAHA.123.22454)
Supplement: Supplementary file 3 [file hyp-81-764-s003.docx]

**Supplemental Material**

**Asymmetric dimethylarginine** enables depolarizing spikes and vasospasm in mesenteric and coronary resistance arteries

Y.Y. Hanson Ng, Kim A. Dora, Hamish A.L. Lemmey, JinHeng Lin, James Alden, Lillian Wallis, Lucy Donovan, Oliver Shorthose, ^§^Fiona C. Leiper, ^§^James Leiper & Christopher J. Garland*

*Author for correspondence:

Department of Pharmacology,

University of Oxford,

Mansfield Road,

Oxford OX1 3QT

e-mail: christopher.garland@pharm.ox.ac.uk

Short title: ADMA and coronary microartery vasospasm

**Expanded Materials and Methods:**

Animals:

Animal use was approved by the University of Oxford ethical committee and complied with the Animals (Scientific Procedures) Act 1986 and European Directive 201/63/EU. Animals were housed in a temperature-controlled environment with a 24-hour light-dark cycle and water *ad libitum.* These studies comply with ARRIVE guidelines.^1^ Male Wistar rats (*circa* 200-310g) were killed as specified by Schedule 1 of the Animals (Scientific Procedures) Act 1986, UK.

Preparation of arteries for myography:

The mesenteric arcade and heart were removed and placed in ice-cold Krebs solution containing (in mmol/L): 1.25 CaCl_2_, 118.00 NaCl, 3.60 KCl, 1.20 MgSO_4_•7H_2_O, 1.20 KH_2_PO_4_ 11.00 glucose, and 25.00 NaHCO_3_ (gassed with 5% CO_2_, 21% O_2_ and balanced with N_2_). A third-order mesenteric artery (external diameter 217-300 μm at 70 mmHg) was dissected free of adherent tissue and a small segment (*circa* 1.5-2 mm) with no visible side branches removed and mounted in Krebs solution in a Mulvany-Halpern wire myograph (Model 610M, Danish MyoTechnology, Denmark). The solution temperature was raised to 37^o^C and allowed to equilibrate for 30 min. Arteries were normalized to a resting tension equivalent to that generated at 90% of the diameter of the vessel at 70 mmHg and left for at least 20 min at resting tension prior to experiment. Artery reactivity was assessed by pre-constriction to phenylephrine (PE, 0.5-3 μmol/L) followed by endothelium-dependent vasorelaxation to acetylcholine (ACh, 0.001-1 μmol/L). Only vessels relaxing >95% were used further. Endothelial cell NO synthesis was inhibited using either 300 µmol/L ADMA or 100 μmol/L L-NAME added to the Krebs buffer. As with mesenteric arteries, intra-septal coronary arteries, *circa* 1.5-2 mm in length and 170-330 µm inner diameter at 80 mmHg (pre myogenic tone), were dissected from surrounding cardiac myocytes and placed in chilled Krebs-buffer. Resistance arteries of this size are defined as pre-arteriolar components of the microcirculation.^2^ A section free from side branches was excised then mounted and normalized in a Mulvany-Halpern wire myograph to a resting tension equivalent to that generated at 90% of the diameter of the vessel at 80 mmHg. Artery reactivity was assessed by ACh, 0.001-1 μmol/L from myogenic tone (characterized by ≥0.5 mN/mm from resting tension).

Measurement of smooth muscle membrane potential:

The vascular smooth muscle membrane potential was measured using sharp glass microelectrodes backfilled with 2 mol/L KCl (tip resistances *circa* 100 MΩ), as previously described.^3^ Smooth muscle membrane potential was recorded through a pre-amplifier (Neurolog system, Digitimer Ltd., U.K.) linked to a MacLab data acquisition system (AD Instruments Model 4e, usually at 100 Hz). Isometric tension and membrane potential were measured simultaneously.

Measurement of VSM intracellular Ca^2+^:

Changes in intracellular [Ca^2+^] were imaged in a confocal wire myograph chamber (Model 120CW, Danish MyoTechnology, Denmark). Artery reactivity was assessed as described above. Viable arteries were then loaded with calcium-sensitive fluorescent indicator CalBryte 520 AM (20650, AAT Bioquest, Pleasanton, USA. 2.5 µmol/L dissolved in DMSO, 0.02% pluronic F-127 and Krebs buffer) for 30 min at 30^o^C, and subsequently incubated in Krebs buffer for 30 min at ~37^o^C to allow de-esterification. Confocal images were obtained using a x40 (0.8 NA, Olympus) water immersion objective and iXON 887 EMCCD camera (Andor Technology, UK) coupled to a Nipkow spinning disk confocal head (CSU22, Yokogawa, Japan) and inverted microscope (IX70, Olympus, Japan). Fluorescence intensity at 513 – 563 nm (excitation 488 nm) was acquired from the bottom surface of arteries at ~35 Hz (430 x 420 pixels) (Andor iQ v3.5, Andor Bioimaging Division, UK). Experiments were performed at 37ºC. Baseline [Ca^2+^]_i_ measurements were established following stabilization of myogenic tone, before addition of 300 µmol/L ADMA at ~37^o^C. Images were stored for offline analysis (iQ version 3.5, Andor Bioimaging Division and MetaMorph version 7.7.4.0, Molecular Devices, San Jose, USA). Data are expressed either as relative fluorescence (F/F_0_) from the full field of view following background subtraction or as frequency of Ca^2+^ events.

Immunohistochemistry:

Viable RMA and RCA were fixed *in situ* in wire myograph chambers with 4% paraformaldehyde for 1 h at room temperature and washed three times (5 min each) with PBS. Fixed arteries were sliced longitudinally into 2 segments using a scalpel and then removed from the wire myograph. Artery segments were incubated for 2 h at room temperature with blocking buffer (1% BSA, 0.5% Triton X-100, 0.05% Tween 20 in PBS), then subsequently incubated overnight with a rabbit polyclonal anti-human DDAH1 antibody (aa 143 – 273; 1:500 dilution, PA5-52278, Invitrogen) in blocking buffer at 4 ^o^C. Arteries were washed the following day before incubation with secondary antibody (goat polyclonal anti-rabbit IgG, 1:1000 dilution, A-11034, Invitrogen) and Hoechst 33342 (1:10,000 dilution, H3570, Invitrogen) for 2 h at room temperature, then washed and placed in mounting medium on glass coverslips with the apical side of endothelial cells face down on the coverslip. Negative control was performed as above, except for primary antibody incubation.

Arteries were excited at 405 and 488 nm with fluorescence intensity at 430-480 and 505-540 nm acquired through a water immersion objective (1.15 NA, 1024 x 1024 pixels; Olympus, UK) using a laser scanning confocal microscope (FV1200; Olympus, UK). Sequential z-stacks through the artery were obtained at 0.5 µm increments at zoom 3.0 using Fluoview Software (FV10-ASW 3.0; Olympus, UK) and first analyzed using Imaris Software (version 8.0.2; Bitplane).

Western blotting:

Rat kidney and liver were excised and immediately snap frozen in dry ice. Tissue was homogenized using CellLytic MT Cell Lysis Reagent (C3228-50ML, Sigma, UK), protease inhibitor (P8430, Sigma, UK), phosphatase inhibitor (P5726, Sigma, UK), Tissue Homogenizing CKMix (P000918-LYSK0-A.0, Bertin Technologies, US) and Minilys Personal Homogenizer (Bertin Technologies, US) and left on ice for 1 h. Homogenate was centrifuged at 4^o^C, 12000 rpm for 20 min. Protein concentration was measured using the Bradford assay.

Equal protein concentrations in loading buffer (Bolt LDS sample buffer, Bolt reducing agent and dH_2_O) were separated by (4-12%) Bolt Bis-Tris Plus Mini Gel (NW04120BOX, Invitrogen, US) in Bolt MOPS SDS running buffer using electrophoresis. Samples were subsequently electro-transferred onto an Immobilon-FL PVDF membrane (IPFL00005, Merck, Germany) in transfer buffer (10% methanol and 0.001% Bolt antioxidant; BT005, Invitrogen, US). Membrane was exposed to blocking buffer (5% milk and 0.001% Tween-20 in TBS buffer, 5% milk-TBST) for 1 h at RT, then incubated with a rabbit polyclonal anti-human DDAH1 antibody (1:500 dilution, PA5-52278, Invitrogen, US) and mouse monoclonal β-actin antibody (1:1000 dilution, AC-15, ab6276, Abcam, UK) in 5% milk-TBST overnight at 4^o^C. Membrane was washed with TBST buffer and blocked for 10 min in 5% milk-TBST, then incubated with IRDye 800CW donkey anti-mouse (925-32212, Li-Cor Biosciences, US) and IRDye 680RD donkey anti-rabbit (925-68073, Li-Cor Biosciences, US) IgG secondary antibodies (at 1:15000 dilution) in 5% milk-TBST for 1 h at room temperature. Western blots were imaged using Li-Cor Odyssey M Imaging System (Li-Cor Biosciences, US) with Empiria Studio Software (v2.2, Li-Cor Biosciences, US).

Data analysis:

Data were analyzed using Microsoft Excel 2011 (Microsoft Corporation) and GraphPad Prism (v10.0, GraphPad Software, USA) software.

For pressurized arteries, the inner diameter was measured using Vasotracker software (v.1.1.0, VasoTracker UK).^4^ Vasorelaxation (wire myograph) and vasodilation (pressure myograph) were expressed as a percentage reversal of tone induced by PE (100% corresponding to the maximal diameter) for RMA and reversal of myogenic tone for RCA. Vasoconstriction/tension was expressed as mN/mm, whereas with pressurized arteries as a percentage of maximum constriction induced by PE for RMA and by myogenic tone for pressurized RCA (100% corresponding to complete closure of artery lumen, inner diameter = 0 µm). Results are summarized as mean ± SEM of *n* replicates, where *n* is the number of individual arteries, each obtained from a separate animal. Statistical analysis performed for each figure panel is indicated in the figure legends, on E_max_ of vasorelaxation/dilation unless otherwise specified. Parametric analysis was performed if half or more of the datasets passed the Shapiro-Wilk normality test. Paired analysis was performed for datasets obtained from the same experiments on the same arteries. Statistical significance is indicated by symbols in each figure legend.

For both membrane potential or tension data from the wire myograph, Fourier transforms were performed on the raw data for quantitative analysis of the waveforms resulting from various treatments. Transforms were carried out with a proprietary MATLAB script to allow for comparison of the frequency responses of the data, including a visual depiction of the aggregated mean power per treatment. Changes in membrane potential, frequency, amplitude and burst frequency were then assessed with proprietary software (LabChart 8).

Previously, we reported tension waveform analysis in mesenteric arteries to characterise PE-induced vasomotion and changes induced by block of NO synthase.^5^ More extensive analysis was attempted here using grouped analysis of either membrane potential or tension. The primary waveform in both occurred around 0.25Hz, with variability reduced by L-arginine. Membrane potential waveforms revealed the appearance of faster components on loss of NO, but this was not apparent with tension. Coronary arteries were less amenable, as they did not develop vasomotion and tension waveforms were flat line, reflecting the fused vasoconstriction/vasospasm. Membrane potential waveforms were more useful, with increased power on loss of NO and a faster primary waveform *circa* 1Hz. Overall, tension waveform was clearly not useful as a surrogate for the recording of membrane potential in future experiments. Analysis of membrane potential waveform may, however, be a useful supplementary approach when comparing arteries from different locations.

Vascular smooth muscle Ca^2+^ imaging data are expressed as the frequency of Ca^2+^ events (Ca^2+^ flashes) observed per second (Hz) within a 30 s duration. For immunohistochemistry, DDAH1 fluorescence intensity at each arterial layer was analyzed either by averaging the fluorescence intensity of five cells per artery segment (for endothelial cell layer) or using the full field of view (for vascular smooth muscle and nerves) using Fiji Software (Version 2.14.0), and subsequently Microsoft Excel and GraphPad Prism.

Materials:

Drug/molecular target nomenclature follows the BJP Concise Guide to Pharmacology.^6^ Phenylephrine (PE; P6126), acetylcholine (ACh; A6625), N_ω_-Nitro-L-arginine methyl ester hydrochloride (L-NAME; N5751), N^G^,N^G^-Dimethylarginine dihydrochloride (ADMA; D4268), N^G^,N^G′^-Dimethyl-L-arginine dihydrochloride (SDMA; 311204), L-arginine monohydrochloride (L-Arg; A5131) and nifedipine (Nif; N7634) were obtained from Sigma (UK). Apamin (Ap; 1652/1), NS6180 (NS; 4864) NNC 55-0396 (NNC; 2268) and levcromakalim (1378) were obtained from Tocris (UK). DDAH1 inhibitor L-257 was provided by Professor James Leiper, University of Glasgow. All stock solutions were prepared using purified (MilliQ quality) water, except for NS6180, NNC 55-0396 and levcromakalim prepared in DMSO, and nifedipine prepared in 100% ethanol. All stocks solutions were prepared at 10^-1^ or 10^-2^ mol/L, and subsequently diluted in Krebs buffer on the day of experiment, except for NS6180 in DMSO to 10^-3^ mol/L. Arteries were pre-incubated with inhibitors for at least 30 min before agonist application, except for apamin and L-257 for 1 h. All drugs were added directly to the bath and mixed with a pipette and continuous gassing.

1. McGrath JC, Lilley E. Implementing guidelines on reporting research using animals (ARRIVE etc.): new requirements for publication in BJP. *Br J Pharmacol*. 2015;172:3189-3193

2. Del Buono MG, Montone RA, Camilli M, Carbone S, Narula J, Lavie CJ, Niccoli G, Crea F. Coronary Microvascular Dysfunction Across the Spectrum of Cardiovascular Diseases: JACC State-of-the-Art Review. *J Am Coll Cardiol*. 2021;78:1352-1371

3. Garland CJ, McPherson GA. Evidence that nitric oxide does not mediate the hyperpolarization and relaxation to acetylcholine in the rat small mesenteric artery. *Br. J. Pharmacol.* 1992;105:429-435

4. Lawton PF, Lee MD, Saunter CD, Girkin JM, McCarron JG, Wilson C. VasoTracker, a low-cost and open source pressure myograph system for vascular physiology. *Front Physiol*. 2019;10:99

5. Smith JF, Lemmey HAL, Borysova L, Hiley CR, Dora KA, Garland CJ. Endothelial nitric oxide suppresses action-potential-like transient spikes and vasospasm in small resistance arteries. *Hypertension*. 2020;76:785-794

6. Alexander SP, Benson HE, Faccenda E, Pawson AJ, Sharman JL, Spedding M, Peters JA, Harmar AJ, Collaborators C. The Concise Guide to PHARMACOLOGY 2013/14: G protein-coupled receptors. *Br J Pharmacol*. 2013;170:1459-1581

**Figures**

**
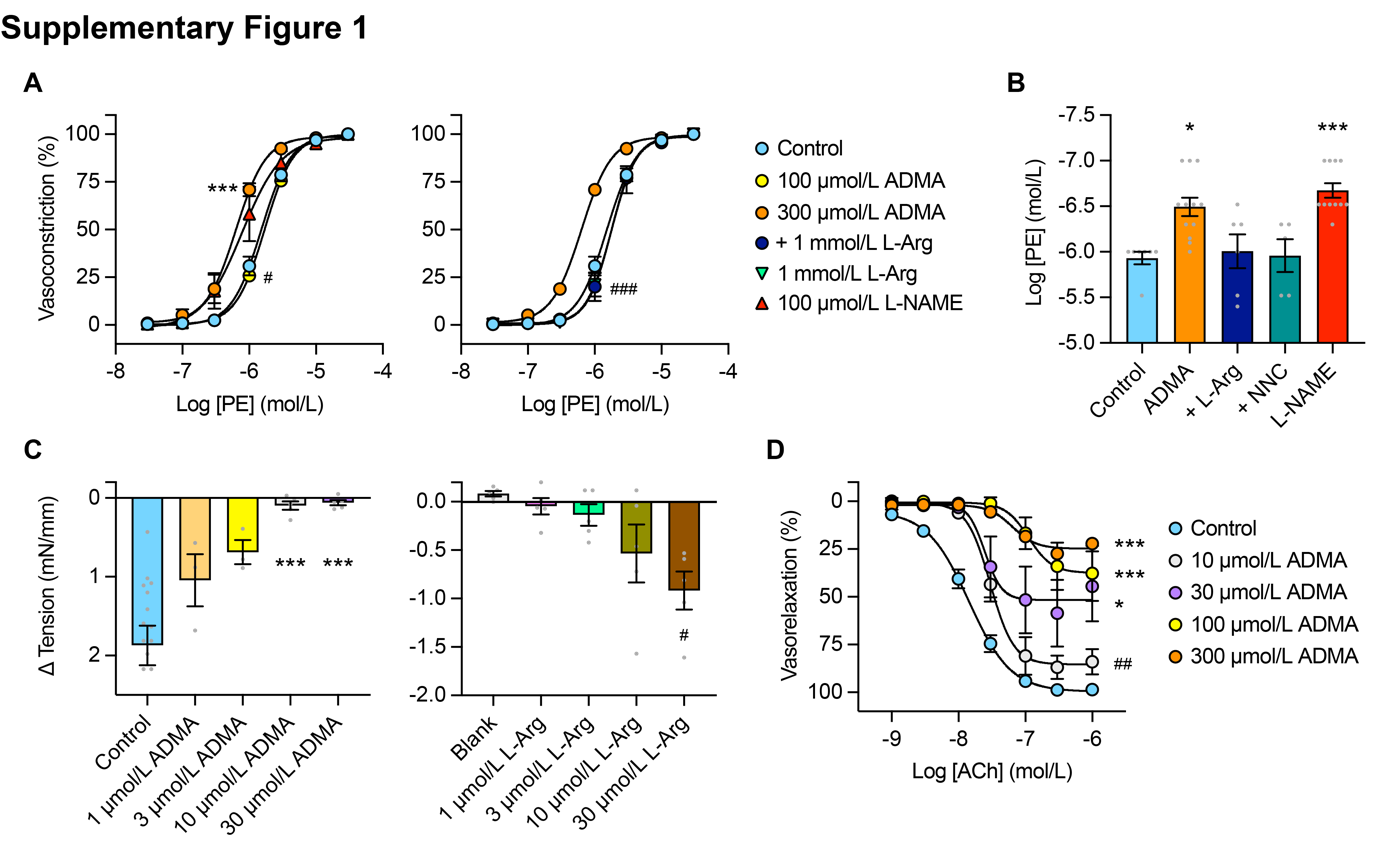
**

**Figure S1:** Effects of ADMA concentration on vasoconstriction and relaxation. **A,** Concentration-dependent vasoconstriction to cumulative [PE] under isometric tension in mesenteric arteries. **Left:** 100 µmol/L ADMA (n=4) did not alter constriction to 1 µmol/L PE compared to control (n=20), whereas 300 µmol/L ADMA (n=9, ***P<0.001 vs control, ^#^P<0.05 vs 100 µmol/L ADMA) augmented contraction, like 100 µmol/L L-NAME (n=6, n.s. vs 300 µmol/L ADMA). **Right:** 1 mmol/L L-arginine (L-Arg, n=7) reversed the effect of ADMA (^###^P<0.001 300 µmol/L ADMA vs + 1 mmol/L L-Arg) but had no effect alone (n=4). One-way ANOVA with Bonferroni’s multiple comparisons. **B**, Concentrations of PE used in Figure 1B-D to generate submaximal constriction in RMA. *P<0.05 vs control, ***P<0.001 vs control; Kruskal-Wallis with Dunn’s multiple comparisons. **C,** Effects of low [ADMA] on myogenic tone in coronary arteries. **Left**: difference in myogenic tone in relation to maximal tone induced by 100 µmol/L L-NAME (0 being maximal). ***P<0.001 vs control; one-way ANOVA with Bonferroni’s multiple comparisons. **Right**: reversal of myogenic tone induced by 10 µmol/L ADMA by increasing concentrations of L-Arg (n=5). ^#^P<0.05 vs blank; RM one-way ANOVA with Bonferroni’s multiple comparisons. **D,** ACh CRCs in the presence of 10 (n=5), 30 (n=5), 100 (n=6), or 300 µmol/L (n=7) ADMA in coronary arteries. *P<0.05 vs control, ***P<0.001 vs control, ^##^P<0.01 vs 300 µmol/L; one-way ANOVA with Bonferroni’s multiple comparisons.

**
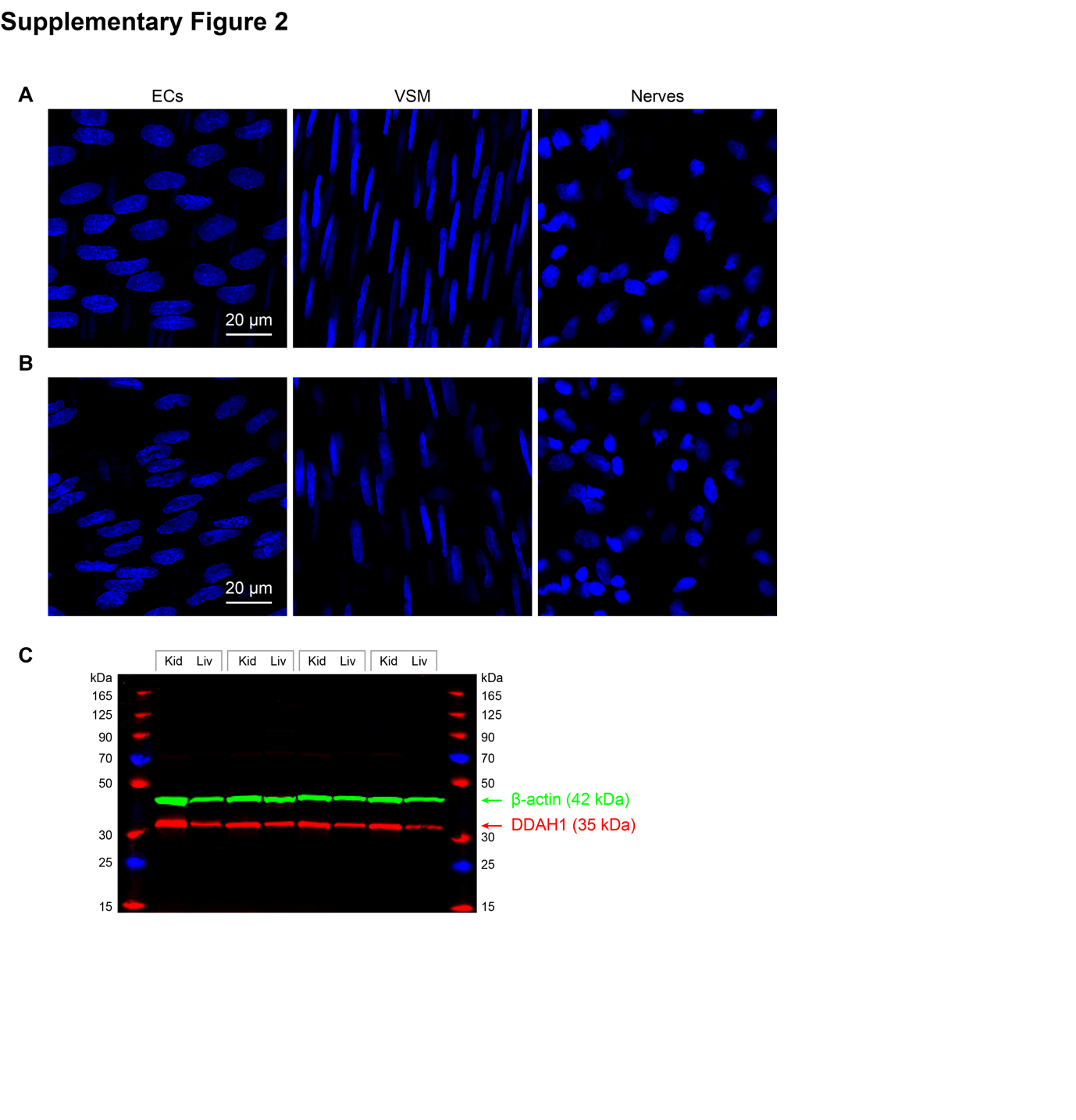
**

**Figure S2:** Negative control and validation of DDAH1 antibody. **A-B,** Negative control of immunohistochemical staining (representative of n = 3 RMA (A) and RCA (B)) with primary DDAH1 antibody omitted. Images were acquired at the same laser settings as images in Figure 4. **C**, Western blot showing DDAH1 expression in rat kidney and liver lysates, with no non-specific bands. β-actin was used as loading control. n = 4.

**
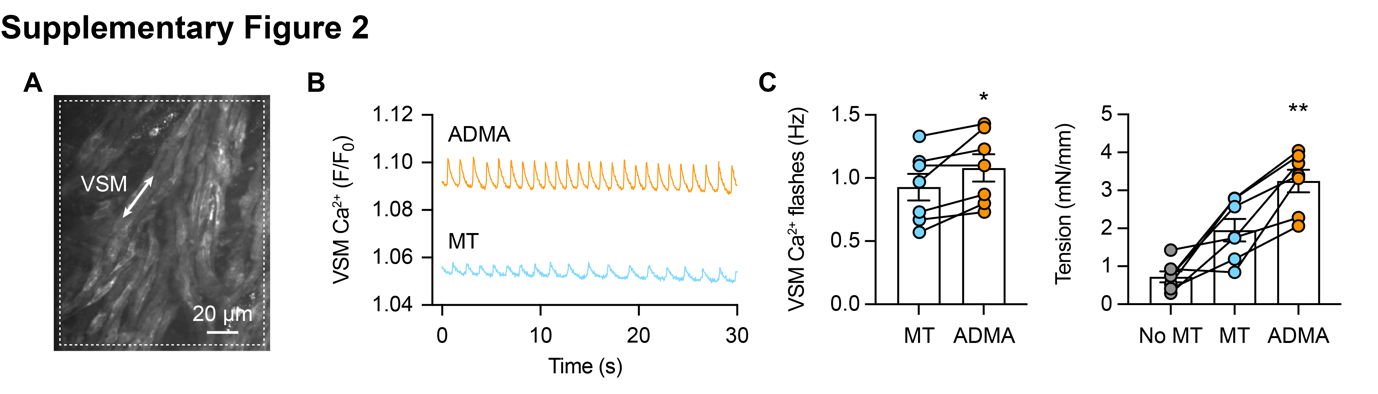
**

**Figure S3:** ADMA increases the frequency of VSM Ca^2+^ flashes and tension in myogenically active coronary artery (RCA). **A**. Representative image of vascular smooth muscle loaded with calcium-sensitive fluorescent indicator CalBryte 520 AM. Dotted white square indicates the area analyzed. Scale bar = 20 µm. **B**, Representative average fluorescence intensity (F/F_0_) traces of Ca^2+^ flashes before (MT) and after the addition of 300 µmol/L ADMA (Movie S1) in the region of artery shown by the dashed box in **A**. **C**, Summary of frequency of Ca^2+^ flashes (left panel) and isometric tension (right panel) in myogenically active coronary arteries before (MT) and after ADMA (n=7, *P<0.05 vs MT, **P<0.01 vs MT; paired t-test). Data represented as mean ± SEM.

**
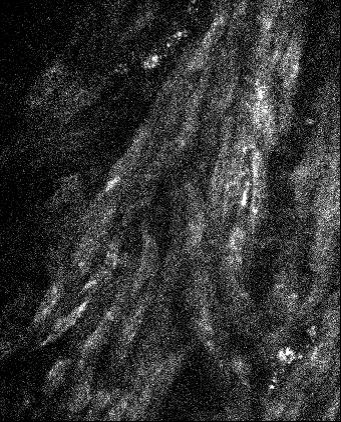
**

**Movie S1:** Ca^2+^ flashes during ADMA in a coronary artery. Movie playback is real time, equivalent to 5.5 s. See separate movie file.

**
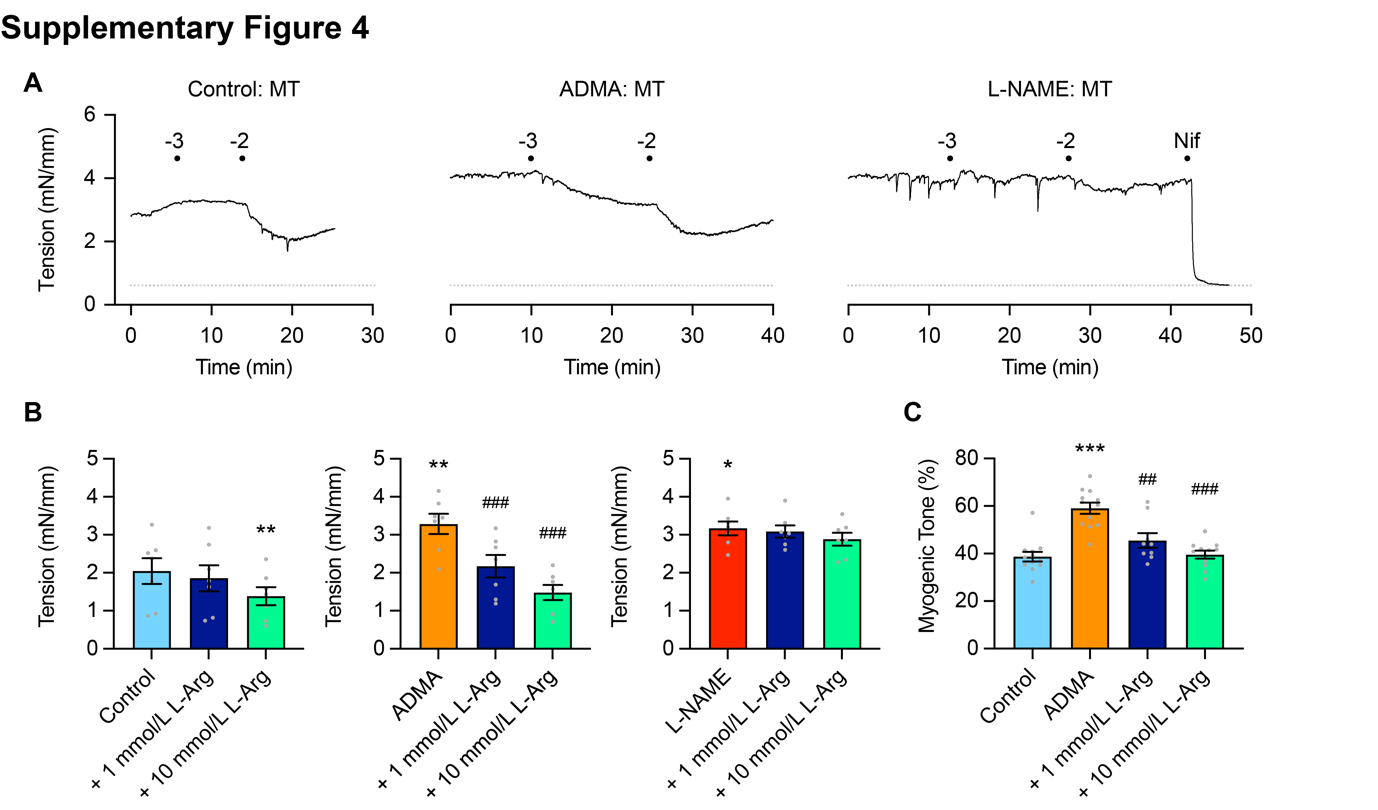
**

**Figure S4:** L-arginine (L-Arg, 1 mmol/L) reversed ADMA-induced vasoconstriction but not control myogenic tone in coronary arteries. **A,** **Left,** vasorelaxation from myogenic tone with 10 mmol/L L-Arg. **Middle,** vasorelaxation to both 1 and 10 mmol/L L-Arg after myogenic tone was increased in the presence of 300 µmol/L ADMA. **Right,** L-Arg failed to reverse vasoconstriction to 100 µmol/L L-NAME, while tone was abolished upon addition of the L-type VGCC blocker, 1 µmol/L nifedipine. **B,** Myogenic tone in the absence (left) and presence (middle) of 300 µmol/L ADMA. Right panel, myogenic tone with 100 µmol/L L-NAME. n=7 in each column, paired experiments. *P<0.05 vs control, **P<0.01 vs control, ^###^P<0.001 vs ADMA; RM one-way ANOVA with Bonferroni’s multiple comparisons. **C,** Myogenic tone as a % of maximum vasoconstriction in pressurized coronary arteries. Control MT (n=12), ADMA (n=12), 1 mmol/L L-Arg (n=9), 10 mmol/L L-Arg (n=11). ***P<0.001 vs control, ^##^P<0.01 vs ADMA, ^###^P<0.001 vs ADMA; one-way ANOVA with Bonferroni’s multiple comparisons.


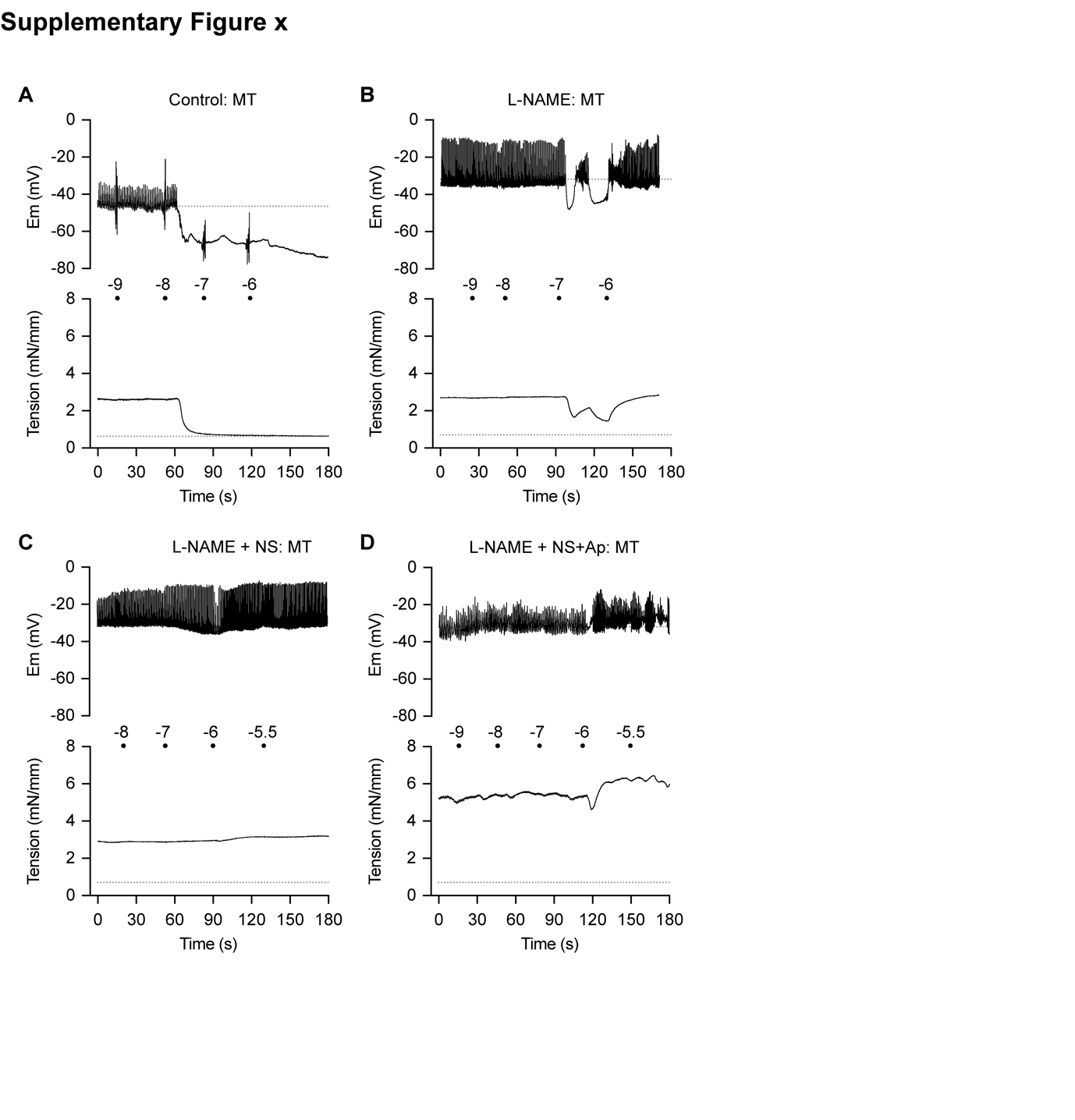


**Figure S5:** Complete inhibition of ACh-mediated hyperpolarization (upper traces) and vasorelaxation (lower traces) by L-NAME and EDH block in coronary arteries. **A,** Control hyperpolarization and vasorelaxation to cumulative [ACh] during myogenic tone (MT). Grey dotted lines, pre-MT. **B,** 100 µmol/L L-NAME enhanced depolarizing spikes and diminished hyperpolarization and vasorelaxation, which became transient. **C, D,** Subsequent additions of 1 µmol/L NS6180 (C) and 0.1 µmol/L apamin (D) completely abolished hyperpolarization and vasorelaxation. Summaries are presented in Figure 6.
